# Artificial intelligence in drug resistance management

**DOI:** 10.1007/s13205-025-04282-w

**Published:** 2025-04-14

**Authors:** Amir Elalouf, Hadas Elalouf, Ariel Rosenfeld, Hanan Maoz

**Affiliations:** 1 Department of Management, Bar-Ilan University, 5290002 Ramat Gan, Israel; 2 Information Science Department, Bar-Ilan University, 5290002 Ramat Gan, Israel

**Keywords:** Antimicrobial resistance, Artificial intelligence, AI applications, Drug resistance management

## Abstract

This review highlights the application of artificial intelligence (AI), particularly deep learning and machine learning (ML), in managing antimicrobial resistance (AMR). Key findings demonstrate that AI models, such as Naïve Bayes, Decision Trees (DT), Random Forest (RF), Support Vector Machines (SVM), and Artificial Neural Networks (ANN), have significantly advanced the prediction of drug resistance patterns and the identification of novel antibiotics. These algorithms have effectively optimized antibiotic use, predicted resistance phenotypes, and identified new drug candidates. AI has also facilitated the detection of AMR-associated mutations, offering new insights into the spread of resistance and potential interventions. Despite data privacy and algorithm transparency challenges, AI presents a promising tool in combating AMR, with implications for improving patient outcomes, enhancing disease management, and addressing global public health concerns. However, realizing its full potential requires overcoming issues related to data scarcity, ethical considerations, and fostering interdisciplinary collaboration.

## Introduction

Antimicrobial resistance (AMR) is a growing global threat, where microorganisms develop resistance to drugs over time in the bacteria, fungi, parasites, viruses, and human cells, making infections more challenging to treat and increasing the risk of severe illness and death. Overuse and misuse of antimicrobial drugs contribute to the rise of drug-resistant pathogens. These overuse and misuse pose challenges to healthcare and exert economic pressure, impacting the success of surgeries and chemotherapy. Addressing AMR requires concerted efforts to preserve the effectiveness of antimicrobial treatments (Tenover and McGowan 2008; Uddin et al. 2021; Salam et al. 2023). As per the World Health Organization (WHO), AMR led to approximately 1.27 million direct global deaths and was linked to about 4.95 million deaths in 2019 (Jonas et al. 2017; Murray et al. 2022). In the United States (US), over 2.8 million antimicrobial-resistant infections arise annually (Brunton and Taylor 2003; Centre for Disease Control and Prevention 2021). A study suggests that around 33,000 individuals succumb each year to infections caused by antibiotic-resistant bacteria, with an impact comparable to the combined burden of influenza, tuberculosis (TB), and HIV/AIDS (ECDC 2018).

Effectively addressing drug resistance is vital for various reasons, including public health, economic factors, and the future sustainability of medicine. Preserving the efficacy of current treatments for infectious diseases is a crucial goal. As microbes evolve and develop drug resistance, effective treatments decrease, underscoring the urgency to impede this process. Drug resistance poses a substantial global public health threat, with resistant strains spreading across borders, causing challenging outbreaks worldwide. Unchecked drug resistance can lead to increased mortality rates and prolonged illnesses, particularly in vulnerable populations. Treatable infections may become untreatable, escalating morbidity and mortality. Drug-resistant infections necessitate longer treatment durations and result in higher healthcare costs, straining healthcare infrastructure and resources. The economic impact is significant, involving extended treatment regimens, more hospitalizations, and the use of costly drugs, contributing to rising healthcare expenses. Moreover, drug resistance intertwines with other health challenges, such as the growing prevalence of non-communicable diseases and the rise in immunocompromised individuals. Effective management of drug resistance is an investment in the health and well-being of future generations, requiring a comprehensive approach that addresses scientific, economic, and public health aspects.

Artificial intelligence (AI) has numerous positive impacts and advancements across various fields, from healthcare and education to industry and beyond. In healthcare, AI aids in faster and more accurate diagnoses, drug discovery, and personalized treatment plans. Similarly, AI is pivotal in managing drug resistance, offering innovative solutions crucial in addressing this global health challenge. The significance of AI in drug-resistance management lies in its ability to expedite and enhance various aspects of the process. AI accelerates drug discovery by swiftly and thoroughly analyzing large datasets, aiding in identifying and designing novel drugs to combat emerging drug-resistant strains. AI tailors treatments by analyzing individual patient data, including genetic information, enabling a personalized approach that optimizes therapeutic outcomes and minimizes the risk of drug resistance. AI algorithms analyze health data patterns to predict the emergence of drug-resistant strains, allowing for early identification and enabling healthcare systems to implement targeted interventions and surveillance measures to mitigate resistance spread. AI tools keep an eye on global health data to spot trends in drug resistance, helping public health agencies and healthcare providers stay ahead of emerging threats for timely and targeted responses. AI makes clinical trials more efficient by finding the right people and predicting risks, speeding up the development of new drugs for a faster response to drug-resistant threats. AI also combines different data types, like clinical and genetic information, giving a complete picture of factors affecting drug resistance and helping create better management strategies.

This review aims to elucidate the intricacies of drug resistance and its effective management through the application of AI. Furthermore, it explores various AI models and algorithms utilized in predicting, optimizing, and managing drug resistance, supplemented by real-time case examples for a comprehensive understanding.

## Incidence of drug utilization

The increased use of drugs can be attributed to factors such as the COVID-19 pandemic, stress, isolation, changes in healthcare policies, legalization of certain substances, increased availability of drugs, and the impact of the pandemic on mental health. The COVID-19 pandemic has caused increased stress and isolation. Pandemic-related stress has led to a 23% rise in alcohol abuse and a 16% increase in drug abuse among prior users. Approximately 13% of Americans have turned to substances to cope with stress, as reported by the Centers for Disease Control and Prevention (CDC) (McLellan 2017; Abramson 2021; Chacon et al. 2021). The pandemic has also prompted changes in healthcare policies related to substance use, facilitating greater access to treatment through initiatives like waiving X waiver programs (Chacon et al. 2021).

Furthermore, the legalization of cannabis in some regions has contributed to heightened daily use and associated health concerns. The increased availability of drugs, including the rise in fentanyl production and distribution, has fueled substance misuse. Mental health impacts of the pandemic are significant, with reports from the Substance Abuse and Mental Health Services Administration (SAMHSA) indicating a 25% increase in opioid overdose deaths in certain areas as of September 2020. These combined factors underscore the complex interplay driving the upward trend in drug use (UNODC 2022).

The global prevalence of drug use has seen a significant increase in recent years. In 2021, more than 296 million people worldwide used drugs, marking a 23% rise compared to the previous decade (UNODC 2023). In addition, the UNODC (United Nations Office on Drugs and Crime) reported that 284 million 15–64-year-olds used drugs worldwide in 2020, a 26% increase over the previous decade (UNDOC 2022). Alongside this, the number of people grappling with drug use disorders surged to 39.5 million in 2021, indicating a substantial 45% increase over ten years (UNODC 2023).

In 2021, the global estimate of people injecting drugs witnessed an 18% increase, reaching a total of 13.2 million individuals (UNODC 2023). Concurrently, young people encountered a substantial 26% surge in drug use, surpassing usage levels observed in the previous generation across multiple countries (UNDOC 2022). Under 35-year-olds are the most likely to seek drug abuse treatment in Africa and Latin America (UNDOC 2022). Meanwhile, in Eastern and Southeastern Europe, along with Central Asia, opioid use disorders stand out as the primary reason prompting individuals to seek treatment (UNDOC 2022).

The US and Canada are facing a significant challenge with a notable rise in overdose deaths, primarily attributed to the non-medical use of fentanyl. Preliminary estimates suggest that there were over 107,000 drug overdose deaths in the US in 2021 (UNDOC 2022). This alarming trend underscores the urgent need for comprehensive measures to address the complexities surrounding drug use and its associated consequences in various parts of the world.

The US has the highest rate of drug use globally, with approximately six percent of people in the country using illegal drugs. Other countries with increased rates of illicit drug use include Greenland, the United Kingdom, and Mongolia, with rates over five percent. However, it is essential to note that drug use rates vary depending on the type of drug and the population studied. For example, Iranians are addicted to opium, including heroin and crystal meth, at higher rates than other countries (2023). A majority of drug use disorders are treated in African and Latin American countries by People under 35 ages. Most people in Eastern and South-East European countries and Central Asia are being treated for opioid use disorders. It is also worth noting that the availability of data on drug use varies by country, and some countries may have higher rates of drug use than reported due to underreporting or lack of data.

According to the literature, drug use is a significant issue in Israel. In 2009, the lifetime prevalence of cocaine use was 2.3%, with last year’s prevalence at 0.9% and the closing month’s prevalence less than 0.5% (EMCDDA 2016). In 2018, the Israel Center on Addiction estimated that one out of every seven Israelis suffers from some form of addiction, with addiction to sex and pornography increasing by 34% and addiction to drugs, mostly marijuana and prescription medicines, up by 15% over the past 5 years (Kashti 2022). In 2020, Israel became the world’s top consumer of potent and addictive drugs per capita, leading the world in prescriptions (Renee Ghert-Zand 2023). The country has implemented a “public health” approach to drug policy, with authorities focusing more on drug dealers than users (Bonny-Noach 2019). To implement and evaluate evidence-based policies and interventions, the Israeli Anti-Drug Authority has established a population survey system (EMCDDA 2016). The country has also adopted harm reduction services, including Needle and Syringe Exchange Programs, Buprenorphine Maintenance Treatment, and Methadone Maintenance Treatment, mainly focused on people who use heroin and people who inject drugs (Bonny-Noach 2019).

## Drug resistance: a growing threat to human beings

Drug resistance poses a growing global threat to health, food security, and development. It can impact individuals of any age, anywhere in the world. While drug resistance is a natural occurrence, the misuse of drugs in humans and animals is hastening this process. A rising number of infections, including pneumonia, TB, gonorrhea, and salmonellosis, are becoming more challenging to treat because the effectiveness of the drugs used against them is diminishing. Drug resistance causes extended hospital stays, increased medical expenses, and higher mortality rates (Prestinaci et al. 2015; Aslam et al. 2018; Ahmed et al. 2021). Globally, AMR caused 1.27 million deaths a year and nearly 5 million deaths in 2019 (CDC 2021). AMR affects people of all ages, socioeconomic statuses, and health conditions (Allel et al. 2020).

**Fig. 1:**
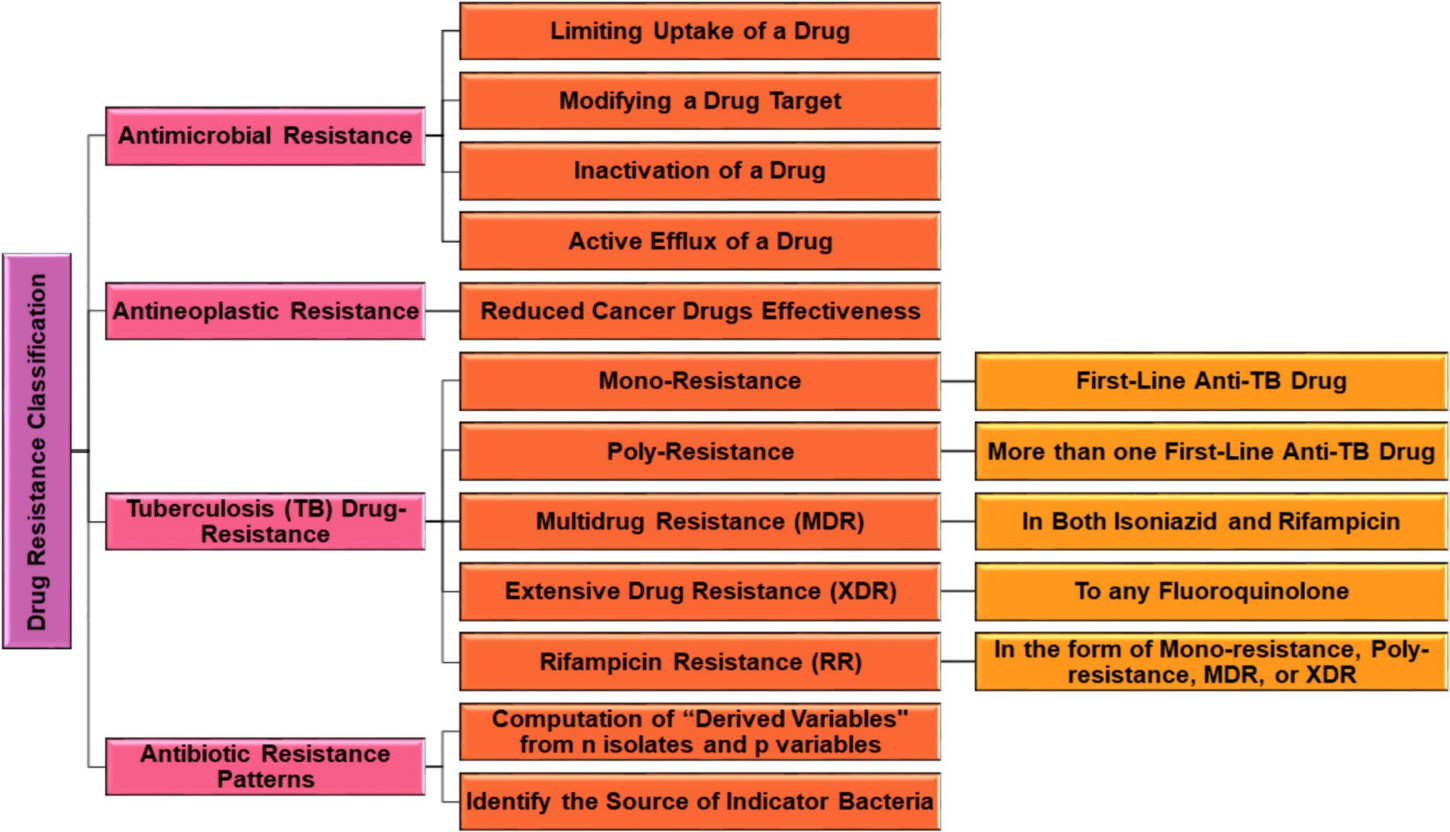
Classification of drug resistance based on the type and extent of resistance. This illustrates various antimicrobial resistance mechanisms, including limiting drug uptake, drug target modification, drug inactivation, and active efflux. It also highlights classifications of antineoplastic resistance due to reduced effectiveness of cancer drugs and drug-resistance TB, categorized into mono-resistance, poly-resistance, multidrug resistance (MDR), extensive drug resistance (XDR), and rifampicin resistance (RR). In addition, it outlines antibiotic resistance patterns involving computational analyses of isolates and variables to identify sources of indicator bacteria

Drug resistance is classified into different categories based on the type and extent of resistance, such as AMR (C Reygaert 2018; Ahmed et al. 2023), antineoplastic resistance, drug-resistance TB (WHO 2021a), and antibiotic-resistance patterns (Harwood et al. 2000), as shown in Fig. 1.

### How does drug resistance develop?

In addition to the misuse of drugs, selective pressure, mutation, gene transfer, inadequate diagnostics, and societal pressure also lead to the development of drug resistance. Antimicrobials create selective pressure for resistant organisms (C Reygaert 2018). For instance, a study evaluated the development of resistance in Gram-negative rods to various antibiotics over 5 years and found statistically significant correlations between the use of certain antibiotics and the development of resistance in specific bacteria (Kolář et al. 2001). This selective pressure can lead to the survival and multiplication of antimicrobial-resistant bacteria, which can then spread their resistance traits to others (Skalet et al. 2010; Centers for Disease Control and Prevention 2021).

Spontaneous mutation is another way bacteria become antibiotic-resistant (C Reygaert 2018). Genetically, bacteria have evolved two major strategies for coping with antibiotics. In the first case, mutations in genes are related to the compound’s action mechanism, while in the second case, foreign DNA is acquired by horizontal gene transfer, coding for resistance determinants (Munita and Hoffman 2016; Ephrem and Sanjay 2022; Muteeb et al. 2023). The “selective pressure” of an antibiotic results in bacteria that acquire a random genetic alteration to survive (Skalet et al. 2010). Bacteria can develop defense mechanisms in response to antibiotics through mutation and selection. For example, several bacteria have developed biochemical pumps to remove antibiotics before reaching their target. Others have evolved to produce enzymes to inactivate the drugs, limiting drug uptake and modifying drug targets (C Reygaert 2018; Zhang and Cheng 2022).

Bacteria can also transfer genes for resistance to other bacteria, making them more resistant to drugs. This transfer can occur through horizontal gene transfer, where bacteria share genes. Bacteria can share antibiotic-resistance genes through direct physical contact, known as conjugation, which allows them to transfer plasmids between themselves (Bello-López et al. 2019; Jian et al. 2021; Tao et al. 2022). In addition, antibiotic-resistance genes can spread via mobile genetic elements, such as in the case of the beta-lactamase gene transferring to *Escherichia coli* (Tao et al. 2022).

Incorrect diagnosis can lead to ineffective treatment, contributing to the development of drug-resistant infections. For instance, inappropriate use of antibiotics can contribute to the development of antibiotic resistance, a global health crisis that threatens the effectiveness of essential antimicrobials (Chinemerem Nwobodo et al. 2022; Salam et al. 2023). Research has shown that 30–50% of antibiotic therapy errors occur due to incorrect indications, choices, or durations (Luyt et al. 2014; Ventola 2015).

Drug-resistant infections pose significant threats, contributing to extended periods of care and recovery, occasionally spanning months and, in severe cases, leading to fatal outcomes (Davies and Davies 2010; Llor and Bjerrum 2014). The repercussions of antibiotic resistance encompass prolonged hospital stays, the emergence of side effects resulting from the use of more potent medications, and an alarming annual incidence of over 2.8 million antimicrobial-resistant infections, causing the death of more than 35,000 individuals (Llor and Bjerrum 2014). It is imperative to adopt responsible antimicrobial usage practices, enhance diagnostic capabilities, and formulate innovative strategies to effectively comprehend and mitigate drug resistance to address the challenges of drug resistance (Kurt Yilmaz and Schiffer 2021).

**Fig. 2:**
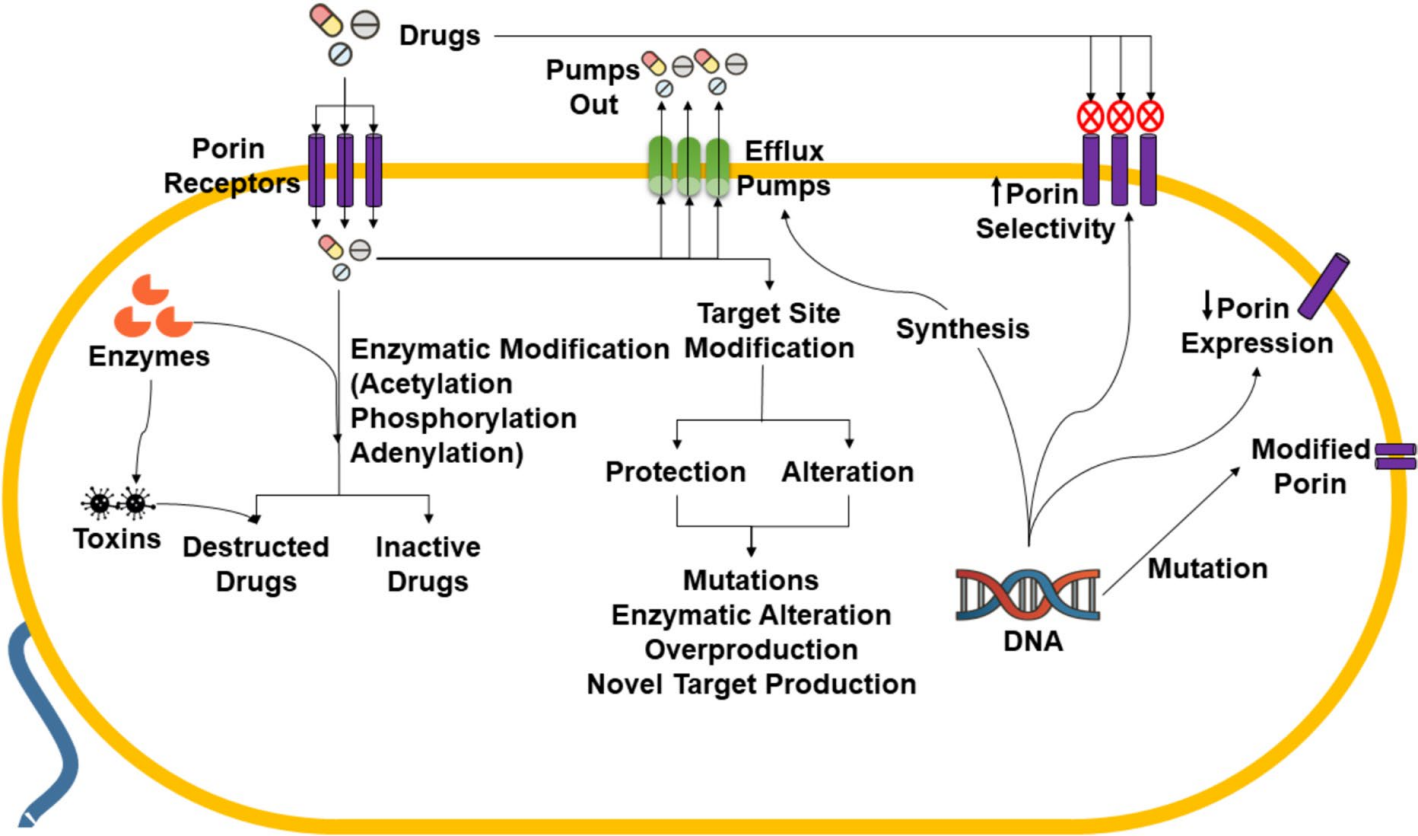
A schematic diagram illustrating diverse mechanisms contributing to developing drug resistance in bacteria. The mechanisms include antibiotic molecule alterations, changes in antibiotic-activating enzymes, decreased membrane permeability, enhanced efflux pump activity, and modifications in antibiotic-active sites. These pathways collectively drive bacterial resistance, as depicted in the schematic. The figure has been adapted with modifications under the Creative Commons CC BY 4.0 License from Ahmed et al. (2023)

All of these contributing factors and mechanisms lead to the development of multiple pathways such as alterations of the antibiotic molecules, alteration of antibiotic-activating enzymes, decrease in membrane permeability and efflux pump activity expression, and alteration in antibiotic-active sites (as represented in schematic Fig. 2) for the drug resistance (Ahmed et al. 2023).

## Drugs resistance management

Coping with drug resistance represents a substantial hurdle in contemporary medical practice due to its adverse impact on the potency of medications for treating various diseases and conditions. Multiple strategies can be implemented to address drug resistance. These include discontinuing the rational use of antimicrobials, adopting combined and integrated therapeutic approaches, implementing preventive and control measures, establishing monitoring and surveillance systems, promoting education and awareness, and allocating resources for research and development.

Prescribing the right drug, dosage, and duration of treatment for a specific infection is crucial to prevent the development of drug-resistant strains (Raymond 2019). Pharmacoepidemiological studies using electronic health records must accurately estimate daily dosage and duration (Zhang et al. 2021). The WHO Collaborating Centre in Oslo assigns a defined daily dose (DDD) to drugs, the assumed average daily maintenance dose for a drug used for its main indication in adults (WHO 2019). Treatment duration is also significant, and an average treatment duration for all subjects reviewed of 23 months was recommended for a specific treatment (Shi et al. 2018).

Combining multiple drugs with different mechanisms of action can help overcome drug resistance, and this approach is beneficial in treating HIV and TB (Raymond 2019). For instance, antibiotic combinations have been developed to combat multidrug-resistant bacteria, and adjuvants that directly target resistance mechanisms have also been developed (Worthington and Melander 2013). In the case of HIV, highly active antiretroviral therapy (HAART) is a combination of drugs that target different stages of the virus’s life cycle, and this approach has been successful in reducing viral load and improving patient outcomes (Wani and Ahmad 2020). In TB treatment, a combination of drugs is used to prevent the development of drug-resistant strains, and the WHO recommends a combination of four drugs for the first 2 months of treatment (Baym et al. 2016).

Implementing interdisciplinary strategies to reduce the carriage and transmission of drug-resistant pathogens is essential for managing resistance (Raymond 2019). The One Health approach highlights the interconnectedness of human, animal, and environmental health, aiming to overcome research bottlenecks, maximize existing research funding, and attract new investments (IACG 2019). This strategy emphasizes collaboration across sectors such as agriculture, healthcare, and environmental management to tackle the emergence and spread of drug-resistant pathogens. Expanding access to diagnostics enhances the treatment of antibiotic-resistant infections, strengthens infection control measures, and improves outbreak detection and response in both healthcare and community settings (States 2014, 2015). Research to improve understanding of environmental factors that enable the spread of resistant genes common to animals and humans can help identify potential interventions to reduce the carriage and transmission of drug-resistant pathogens (Bengtsson-Palme et al. 2018; Lepper et al. 2022). Implementing interdisciplinary strategies to improve infection prevention and control in healthcare facilities, farms, schools, households, and community settings can help reduce the spread of drug-resistant pathogens (O. Popoola 2023).

Focusing on infection prevention and control practices, such as hand hygiene, proper use of personal protective equipment, and vaccination, can help reduce the spread of drug-resistant infections. For example, good hygiene practices, infection prevention, and control procedures can minimize the spread of antibiotic-resistant bacteria (Maillard et al. 2020). The CDC’s Core Infection Prevention and Control Practices for Safe Healthcare Delivery in All Settings outlines a core set of infection prevention and control practices applicable across healthcare settings. According to the National Action Plan for Combating Antibiotic-Resistant Bacteria (2015), improving access to diagnostics is crucial for enhancing the treatment of antibiotic-resistant infections, strengthening infection control measures, and enabling more effective outbreak detection and response in both healthcare and community settings. In addition, the WHO highlights the importance of adequate access to clean water, sanitation, and hygiene in healthcare facilities, farms, schools, households, and community settings to prevent the spread of drug-resistant pathogens (IACG 2019).

Regularly monitoring drug-resistance patterns and trends is essential to identify areas where resistance is becoming a problem and to guide the development of new strategies to combat it (Bengtsson-Palme et al. 2023). Different approaches have been used to monitor and observe drug resistance, such as antibiograms, the National AMR Monitoring System (NARMS), and the Global AMR Surveillance System (GLASS). Antibiograms help monitor trends in pathogens’ phenotypic resistance to different drugs. They are invaluable in clinical and public health settings for tracking resistance patterns and guiding treatment decisions (Buckley and Palmer 2022). NARMS is a US public health surveillance system that monitors AMR among enteric bacteria from humans, food, and animals’ food. It tracks resistance patterns and trends to understand and prevent the transmission of antimicrobial-resistant bacteria (CDC 2018). GLASS is a significant step forward in monitoring AMR at a global level. It allows real-time monitoring of AMR trends and the detection of emerging resistance, providing essential data to inform public policy and interventions (Dimegni and Lawson 2022). Public health authorities and healthcare providers can make informed decisions about treatment guidelines, infection control measures, and developing new antimicrobial strategies to combat drug resistance by monitoring resistance patterns and trends.

Raising awareness about drug resistance among healthcare professionals, patients, and the general public can help promote responsible use of antimicrobials and reduce the development of drug-resistant strains. A study published in PLOS One demonstrated the effectiveness of increasing high school students’ awareness about the consequences of antibiotic resistance and the importance of responsible antibiotic use (Fonseca et al. 2012). Research conducted among medical students in Kerala, India, and Riyadh, Saudi Arabia, emphasized the importance of educating future healthcare professionals to create awareness and promote responsible antibiotic use (Reena and Ittyachen 2022; Almutairi et al. 2023). The U.S. National Action Plan for Combating Antibiotic-Resistant Bacteria includes strategies to expand public education and awareness programs to promote the appropriate use of antibiotics and prevent the emergence of resistant infections (2015). Implementing educational interventions and raising awareness at various levels can positively influence AMR knowledge, perceptions, and behaviors, ultimately contributing to the responsible use of antimicrobials and reducing drug-resistant infections.

Investment in research and development (R&D) is essential for overcoming the challenges of drug-resistant pathogens. Global Antibiotic Research and Development Partnership (GARDP), created by the WHO and the Drugs for Neglected Diseases Initiative (DNDi), aims to accelerate the development of new treatment options for drug-resistant infections while ensuring access and stewardship (WHO 2021b). The Joint Programming Initiative on AMR (JPI AMR) initiative funds basic and preclinical research that addresses the human health R&D challenge related to AMR (Joint Programming Initiative on Antimicrobial Resistance 2015; Kelly et al. 2016). The U.S. National Action Plan for Combating Antibiotic-Resistant Bacteria emphasizes the need to stimulate R&D for new antibiotics and diagnostic tools to combat AMR (2015). Despite antibiotic resistance being a major global health threat, the antibiotic research pipeline remains dry due to various scientific, economic, and regulatory challenges. Efforts have been made to reinvigorate the antibiotic pipeline, and there is a continual need for an optimal and sustainable arsenal of effective antibiotics (Wasan et al. 2023). These examples demonstrate the importance of ongoing investment in R&D to develop new drugs, drug combinations, and diagnostic tools, which are essential for effectively addressing and managing drug-resistant pathogens.

### WHO rules and guidelines for prevention and control of drug resistance

The WHO has established comprehensive rules and guidelines to prevent and control drug resistance. These guidelines encompass various strategies, including the rational use of antimicrobials, such as antibiotics, antivirals, antifungals, and antiparasitic drugs, to mitigate the development of drug-resistant strains. The WHO advocates for implementing surveillance systems to monitor the emergence and dissemination of drug-resistant strains, emphasizing the importance of early detection. In addition, infection prevention and control measures, such as hand hygiene, proper use of personal protective equipment, and vaccination, are recommended to curb the spread of drug-resistant infections. Antimicrobial stewardship programs are encouraged to promote judicious antimicrobial use and reduce the emergence of drug resistance. Furthermore, the WHO underscores the significance of ongoing investment in research and development to innovate new drugs, drug combinations, and diagnostic tools for detecting and monitoring drug resistance. Adhering to these guidelines makes it possible to prevent and control drug resistance effectively, ensuring the sustained efficacy of medications in treating infections and cancers (Raymond 2019; WHO 2021a; Kurt Yilmaz and Schiffer 2021).

### Policies for drug resistance by health professionals and policymakers

Health professionals and policymakers can implement policies to address drug resistance (Van Katwyk et al. 2019; Al-Haboubi et al. 2020; Hannah and Baekkeskov 2020). The CDC provides guidelines for preventing and controlling multidrug-resistant organisms (MDROs) in healthcare settings. These guidelines emphasize the importance of infection prevention, accurate diagnosis and treatment, prudent use of antimicrobials, and prevention of transmission (Centers for Disease Control and Prevention 2016). The Society for Healthcare Epidemiology of America and the Infectious Diseases Society of America have also issued guidelines for the prevention of AMR in hospitals. These guidelines stress the significance of appropriate antimicrobial stewardship, infection control programs, and the optimal selection, dose, and duration of treatment to prevent or slow the emergence of resistance among microorganisms (Shlaes et al. 1997). The WHO advocates for the rational use of antimicrobials, surveillance and monitoring of drug resistance, infection prevention and control measures, antimicrobial stewardship, and continued investment in research and development to address drug resistance (Tenover and McGowan 2008). These policies and guidelines focus on responsible antimicrobial use, infection control, and surveillance to prevent and control drug resistance, highlighting the need for a multidisciplinary approach involving healthcare professionals and policymakers (Shlaes et al. 1997; Tenover and McGowan 2008; Centers for Disease Control and Prevention 2016).

## Emerging challenges in drug resistance management

The emerging challenges in drug resistance management include the evolutionary pressure, alternative resistance mechanisms (decreasing the effective drug concentration, eliminating "persister" cells, and genetic changes), global impact and spread of resistance, and accelerating factors like overuse and misuse of antimicrobials, inappropriate use, sub-therapeutic dosing, and patient noncompliance. Table 1 comprises the central points related to the emerging challenges in drug resistance management.

**Table 1:** Main emerging challenges in drug resistance management

Main challenges	Key points	References
Evolutionary pressure	Survival-driven evolution leads to drug resistance High prevalence in oncology and infectious diseases Pathogens evolve to resist antimicrobials Impact across different fields, including cancer treatment AMR is a global crisis	(Read et al. 2011; Merker et al. 2020; Kurt Yilmaz and Schiffer 2021; Pressley et al. 2021)
Alternative resistance mechanisms	Involves mechanisms like decreased drug concentration, elimination of “persister” cells, and genetic changes Resistance mechanisms in bacteria include limiting uptake, drug target modification, inactivation, and active drug efflux Understanding these mechanisms is crucial for developing effective antimicrobial drugs	(Baym et al. 2016; C Reygaert 2018)
Lack of evolution-proof drugs	No drug is evolution-proof New drugs must be used sustainably Evolution of drug resistance in bacteria affects various medical areas Multidrug resistance may require sequential accumulation of resistance Understanding mechanisms for better treatment options is essential	(Baym et al. 2016; Raymond 2019; Chifiriuc et al. 2022; Chinemerem Nwobodo et al. 2022; Coque et al. 2023)
Spread of resistance	Spread through transmission or spontaneous evolution Integrated approaches needed to reduce carriage and transmission of multi-resistant pathogens Bacteria evolve resistance through spontaneous mutation and horizontal gene transfer Implementing strategies to address both evolution and transmission is essential	(Baym et al. 2016; Bello-López et al. 2019; Gabaldón 2023)
Global impact	AMR has a global impact Rising bacterial resistance poses a threat to common antibiotics Estimated 4.95 million deaths globally in 2019 due to drug-resistant infections Antibiotic resistance is a top threat to public health, causing significant infections and deaths annually	(Centers for Disease Control and Prevention (CDC) 2021; Murray et al. 2022)
Accelerating factors	Overuse and misuse of antimicrobials accelerate drug resistance Factors include inappropriate use, sub-therapeutic dosing, and patient noncompliance Over-prescription of antibiotics, especially in developed countries, is a leading factor Overuse of antibiotics in agriculture and the spread of infectious diseases contribute to resistance Addressing these factors is crucial to preserving antimicrobial effectiveness	(Knobler et al. 2003)

### Antibiotic resistance: biggest threats to global health and food security

Antibiotic and antiviral resistance pose significant threats to global health and food security. The emergence of drug-resistant strains of bacteria, viruses, fungi, and parasites is a growing concern, impacting healthcare, food production, and life expectancy. The WHO has identified AMR as one of the most severe global public health threats affecting human health and food security (Salam et al. 2023). The overuse and misuse of antibiotics in human and animal health and agriculture have contributed to the rise of drug-resistant strains. The misuse of antibiotics, inappropriate choices, inadequate dosing, and poor adherence to treatment guidelines have been identified as factors contributing to increased antibiotic resistance (Prestinaci et al. 2015). The modern travel of people, animals, and goods means that antibiotic resistance can quickly spread across borders and continents, emphasizing the need for collaborative, coordinated efforts to slow its development and spread (Aslam et al. 2021a; Ahmad et al. 2023). Concerted efforts are needed to address the antibiotic resistance crisis by implementing new policies, renewing research efforts, and pursuing steps to manage the situation. Strategies to minimize antibiotic resistance include using antibiotics prudently based on guidelines, controlling their use in food, animals, and educating patients, the public, and healthcare professionals about the responsible use of antibiotics (Lee et al. 2013; Ventola 2015).

### Managing drugs resistance with vaccine

Vaccination plays a crucial role in combatting AMR. Unlike antibiotics, vaccines do not exert the same evolutionary pressure on pathogens, making vaccine resistance less of a concern than drug resistance (Micoli et al. 2021). Vaccines effectively prevent resistant infections and reduce the need for antibiotics, thereby decreasing the emergence and spread of AMR (Alghamdi 2021). They can also induce herd immunity, indirectly protecting unvaccinated individuals and reducing the circulation of resistant strains in vaccinated populations (Jansen et al. 2018; Alghamdi 2021). Vaccines are being developed to prevent infections with Gram-negative bacteria, including *E. coli* and *K. pneumoniae*, which pose significant concerns due to their high resistance levels to third-generation cephalosporins and carbapenems (Jansen et al. 2018). Vaccines offer numerous benefits in combating AMR. Even so, the diversity of bacteria, the variety of potential antigens, and the ability to cause various infections with different pathogenic mechanisms can pose challenges to the development of vaccines (Mullins et al. 2023).

Herd immunity is a strategy that can mitigate the propagation of drug-resistant pathogens and reduce the need for antibiotics, contributing to the mitigation of AMR (Suryawanshi and Biswas 2023). Vaccines can lessen the prevalence of resistant pathogens and prevent resistant strains from spreading (Micoli et al. 2021). Herd immunity can protect vulnerable groups, including those at higher risk of infection (Suryawanshi and Biswas 2023). Vaccines can be used prophylactically, reducing the likelihood of resistance-conferring mutations emerging (Micoli et al. 2021). Herd immunity requires a substantial proportion of the population to be vaccinated, lowering the overall amount of virus able to spread in the whole population. Achieving herd immunity with safe and effective vaccines makes diseases rarer and saves lives (She et al. 2022).

**Fig. 3:**
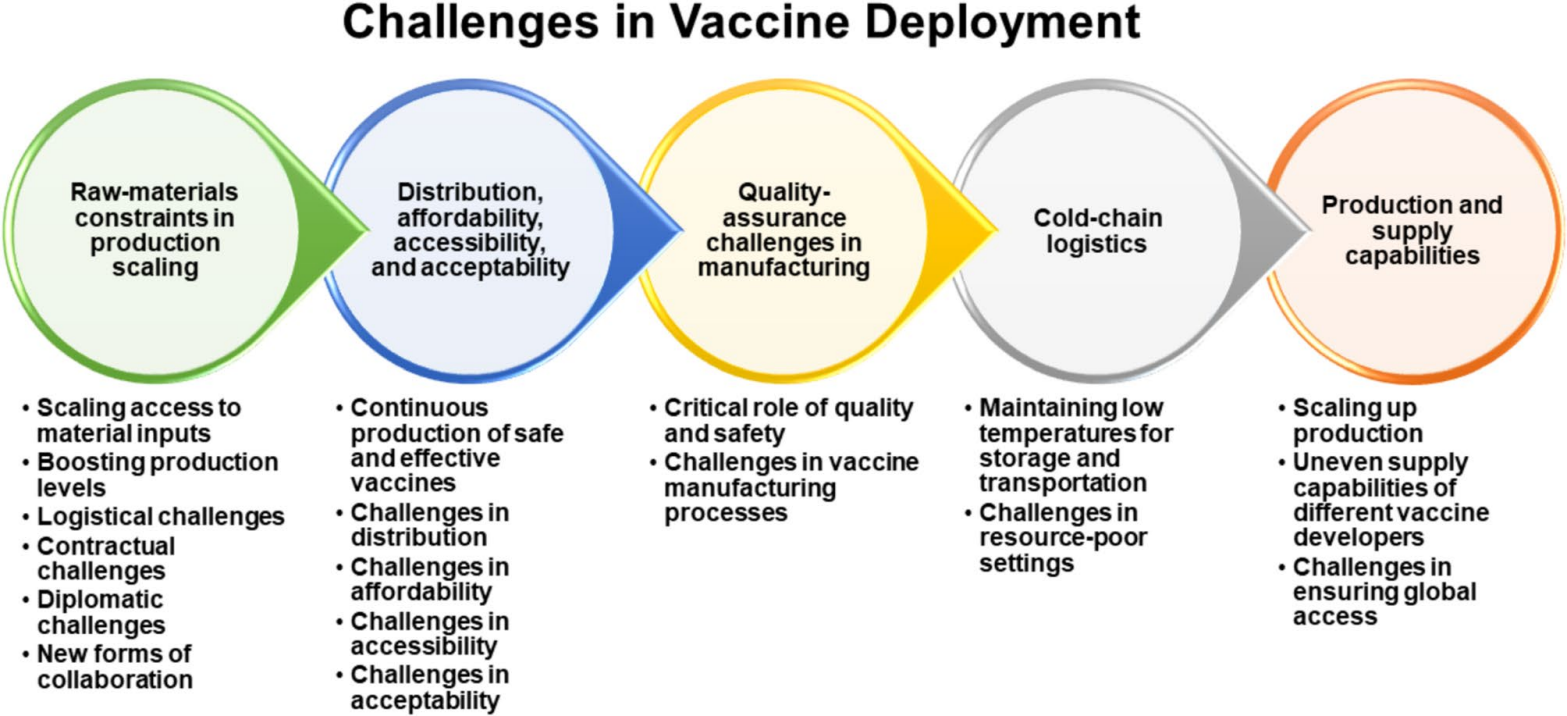
Challenges in vaccine deployment. This highlights the multifaceted challenges associated with vaccine development and distribution, including raw-material constraints, scaling production, and addressing logistical, contractual, and diplomatic hurdles. It emphasizes the importance of new forms of collaboration and ensuring vaccines are distributed affordably, accessibly, and acceptably. Additional challenges include maintaining quality assurance in manufacturing, ensuring safety, addressing cold-chain logistics for storage and transportation, and overcoming barriers in resource-poor settings. The figure also underscores issues in production scalability, uneven supply capabilities among developers, and the critical need to ensure equitable global access to vaccines

Challenges in vaccine deployment include the need for large-scale catch-up to achieve population-level protection against vaccine-resistant pathogens and the potential for vaccine resistance to involve other phenotypes, such as immune suppression and faster replication, which could cause more severe disease in unvaccinated individuals. Developing vaccines for resistant pathogens and achieving broad vaccine coverage pose logistical and research challenges (Agrawal et al. 2021; Wouters et al. 2021; Yarlagadda et al. 2022; She et al. 2022; Suryawanshi and Biswas 2023), as shown in Fig. 3.

### Society engagement and education about drug resistance

**Fig. 4:**
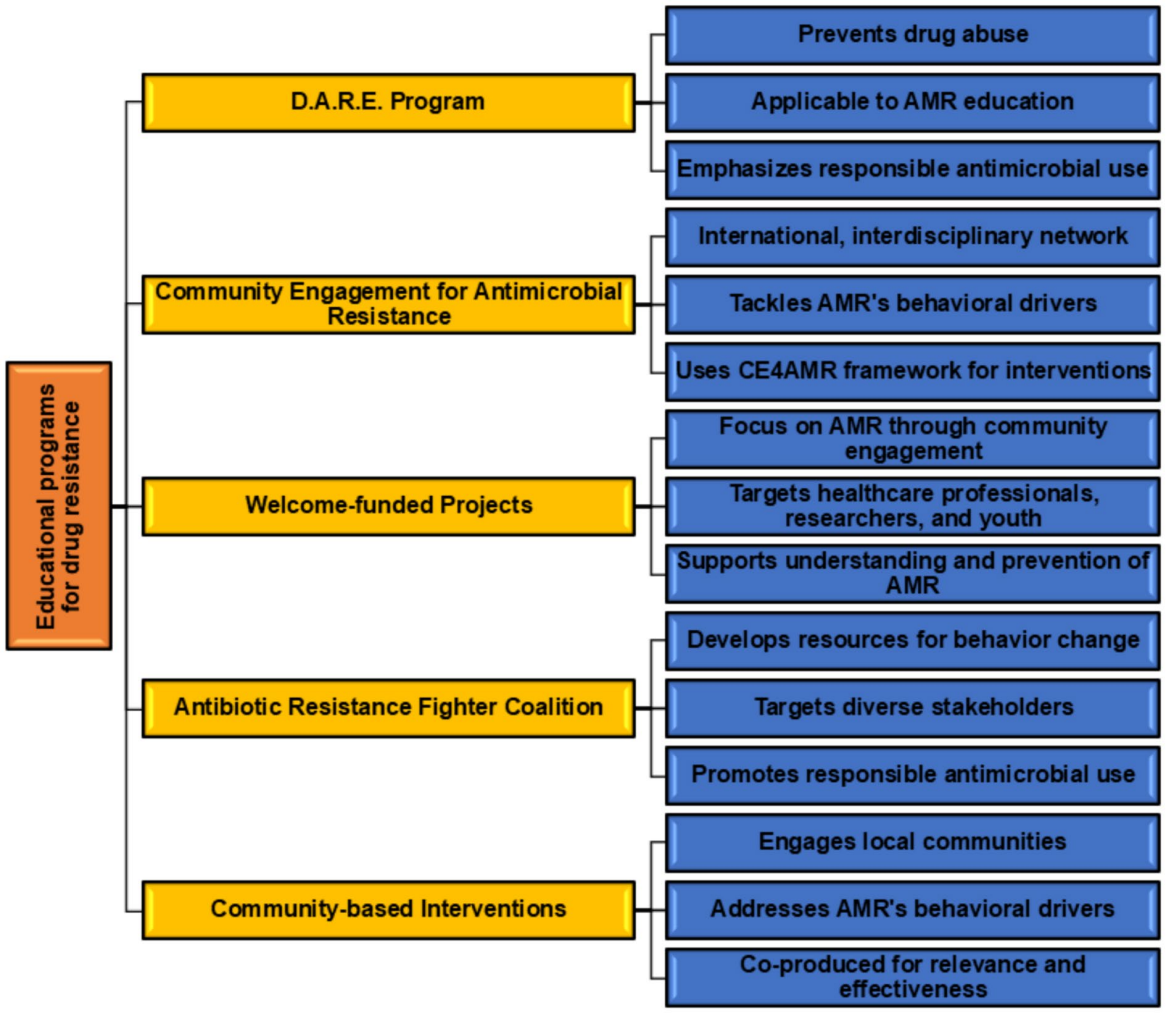
Programs, initiatives, and strategies combatting AMR. This illustrates various global and community-based efforts aimed at addressing AMR. Key elements include educational programs like the D.A.R.E. Program, adapted to promote responsible antimicrobial use, and community engagement initiatives, such as the CE4AMR framework, targeting AMR’s behavioral drivers. Highlighted projects include Welcome-funded programs, which focus on AMR awareness among healthcare professionals, researchers, and youth, and the Antibiotic Resistance Fighter Coalition, which develops resources for behavior change and responsible antimicrobial use. In addition, community-based interventions emphasize locally relevant, co-produced strategies to tackle AMR effectively

Society engagement and education about drug resistance are essential for addressing the challenges posed by AMR. Various programs and initiatives have been implemented to educate and inform the public about the importance of combating AMR and promoting the responsible use of antimicrobials (Ennett et al. 1994; World Health Organization 2014; Mitchell et al. 2022, 2023a, b), as shown in Fig. 4. Programs like Drug Abuse Resistance Education (D.A.R.E.) strive to deter drug abuse and related behaviors. Community engagement endeavors, exemplified by initiatives like Community Engagement for AMR (CE4AMR), focus on tackling behavioral factors contributing to AMR through participatory methods (Mitchell et al. 2023b). These efforts are critical for fostering responsible behavior and decision-making concerning drug use and AMR at the community level. Health professionals and policymakers play vital roles in combating AMR, employing strategies such as optimizing antibiotic use through surveillance and education, integrating AMR awareness into professional training, regulating the antibiotic industry while fostering innovation, implementing national action plans to prioritize AMR, collaborating globally through organizations like WHO, and engaging communities in developing effective interventions. These efforts aim to reduce AMR emergence and spread, ensuring the continued effectiveness of antimicrobial treatments (Shelke et al. 2023).

## Role of AI in drug resistance management

AI has been applied in various aspects, such as designing, discovering, predicting, monitoring, and investigating for AMR management. It can create personalized treatment plans to match each patient’s genetic traits and disease characteristics. Recent scientific studies show that AI has been used to design new antibiotics and generate synergies of drug combinations. AI applications have been commonly used in developing new antibiotics and exploring synergistic antibiotic combinations against AMR using predictive AMR models and the rational use of antibiotics (Lv et al. 2021; Rabaan et al. 2022; Ali et al. 2023). Healthcare policymakers and providers can decide which drugs to prescribe by analyzing patterns in data on antimicrobial use and resistance (Ali et al. 2023). Deep learning approaches have also expanded our antibiotic arsenal by discovering structurally distinct antibiotics (Stokes et al. 2020).

There are several examples of AI being used to design new antibiotics. The most recent one is the MIT researchers’ discovery of a class of compounds that can kill a drug-resistant bacterium such as methicillin-resistant *Staphylococcus aureus* that causes more than 10,000 deaths in the US annually (Trafton 2023; Wong et al. 2023). Another example is the company Insilico Medicine, which uses AI to design new compounds that can be used to treat cancer, other diseases, and bacterial infections (Matt Nicholson 2023). The company has developed an AI-powered platform called GENTRL to generate new compounds with antibiotic activity. In addition, deep learning approaches have expanded our antibiotic arsenal by discovering structurally distinct antibiotics (Anne Trafton 2020). While specific names of combined drugs synthesized using AI for resistant organisms are not provided in the search results, the use of AI in developing new antibiotics and exploring synergistic antibiotic combinations is well-documented (Rabaan et al. 2022). Another study reported the discovery of halicin, the first powerful antibiotic discovered using AI (Marchant 2020).

The efficacy of AI in developing antimicrobial drug molecules for addressing drug-resistant infections encompasses the rapid application of AI in diverse aspects of antimicrobial peptides, drug development, essential oils, spanning small molecules, phage therapy, and resistance mechanism prediction (Talat and Khan 2023). Developing new antibiotics for pathogens on the WHO priority list is becoming increasingly challenging due to rediscovery problems and inadequate new drug development. The importance of AI in identifying targets, doing dynamic modeling, designing and synthesizing peptides, and evaluating SARs and STRs is underscored, as well as repurposing drugs (Talat and Khan 2023). Noteworthy advancements in AI’s contribution to discovering small molecule antibiotics and antimicrobial peptides are elucidated, emphasizing aspects. For instance, AutoMolDesigner, an AI-based open-source platform, is designed to accelerate antibiotic discovery by predicting antimicrobial activity, determining drug-likeness traits, antimicrobial compound representation, AMR, and de novo molecular design (Shen et al. 2024).

In addition, AI algorithms are recognized for their capability to generate proteins, peptides, nucleic acid biologics, and immunotherapeutics with enhanced stability, binding affinity, and pharmacokinetics. The study concludes that AI has significant potential to accelerate antibiotic discovery and development, serving as a valuable tool to address the escalating issue of drug-resistant infections (Melo et al. 2021; Vora et al. 2023; Liu et al. 2024).

Researchers have recently been developing ML algorithms that can accurately predict antibiotic resistance. ML algorithms analyze data on the use of antimicrobial drugs and their resistance to predict which microorganisms will develop resistance to certain drugs (Farhat et al. 2023). These algorithms can encourage healthcare providers and policymakers to decide which drugs to use (Ali et al. 2023). ML algorithms may also assist clinicians in predicting the emergence of AMR, allowing them to use more targeted antibiotics and reduce the need to treat the same infection with multiple antibiotics (Sakagianni et al. 2023). ML methods are trained on numerous patient records and AMR measures to predict resistance against different antibiotics, helping avoid treatment failures and enabling more targeted antibiotics (Sakagianni et al. 2023).

Study findings in two Vietnamese hospitals evaluated the accuracy of ML models for predicting AMR bacteria and resistance to antibiotics. To predict antibiotic resistance in ICU patients in Vietnam, XGBoost, LightGBM, and RF were found to be the best-performing ML models. Researchers should consider various predictors of resistance in future research, such as residency location, antibiotic use outside the hospital, and microbiome composition. Based on the results of this study, ML could be used to predict antibiotic resistance in Vietnamese ICU patients (Quoc et al. 2023).

AI can inspect and monitor AMR, helping healthcare providers and policymakers decide which drugs to use (Ali et al. 2023). AI can help quickly identify potential antibiotics by screening known compounds and predicting their resistance patterns. AI can facilitate target identification and dynamic modeling, which can help design better antibiotics. AI can leverage known genomic data to predict the potential sites of resistance related to enzymes’ functions, setting the groundwork for developing better antibiotics (Liu et al. 2024). ML models can monitor AMR, helping healthcare providers and policymakers decide which drugs to use (Ali et al. 2023).

**Fig. 5:**
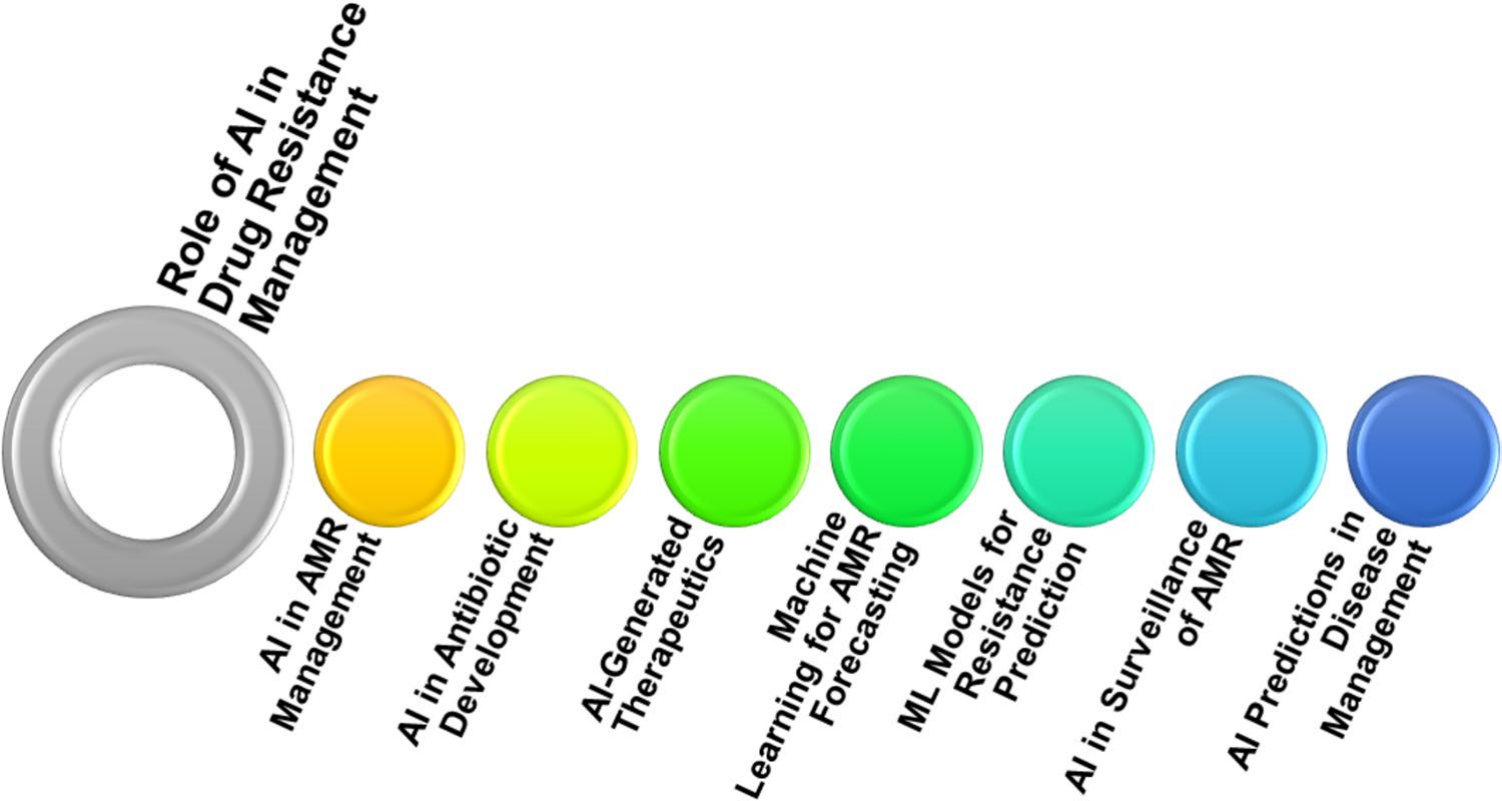
Role of AI in drug resistance management. This highlights the multifaceted applications of AI in addressing antimicrobial resistance (AMR), including its use in AMR management, antibiotic development, and AI-generated therapeutics. Machine learning (ML) models play a pivotal role in forecasting resistance patterns and predicting resistance mechanisms. AI also enhances the surveillance of AMR and supports accurate predictions for disease management, showcasing its transformative potential in combating drug resistance

AI is proving to be especially valuable in predicting drug resistance patterns, a critical aspect of managing diseases. Predicting drug resistance patterns helps healthcare professionals foresee which patients might develop resistance to specific medications. This knowledge guides treatment decisions and helps stop the spread of drug-resistant strains. AI’s strength lies in its ability to process vast amounts of data and uncover patterns that might be hidden from human researchers. This merit makes its predictions about drug resistance more accurate and efficient. It can transform disease management by tailoring treatment plans to each patient’s genetic makeup and characteristics. It can make personalized treatment plans based on each person’s unique genes and disease. AI is especially good at predicting drug resistance, which helps us know which medicines will work. Figure 5 presents the role of AI in drug resistance management.

### The challenges in using AI for drug resistance management

AI has shown great promise in drug resistance management, but several challenges must be addressed. The role of AI in drug resistance management faces significant challenges—Table 2 shows the challenges in harnessing AI for drug resistance management. First, data on antimicrobial use and resistance, crucial for successful AI models, are often incomplete and inconsistent, hampering accurate model development. Second, complexity in interpreting AI models poses a second challenge, hindering transparency in decision-making. Third, generalizability is limited, and applying AI models across different settings is challenging. In addition, regulatory and ethical issues must be addressed, including data privacy and informed consent. Finally, the high cost and lack of universal infrastructure hinder the widespread implementation of AI models in drug resistance management (Rabaan et al. 2022; Ali et al. 2023; Liu et al. 2024).

**Table 2:** Challenges in hanessing AI for drug resistance management

Challenge	Description	Examples	References
Data quality and availability	Data quality and availability are crucial for successful AI models in drug resistance management. Incomplete, inconsistent, and difficult-to-access data hinder model accuracy	ML models analyzing patterns in incomplete data—difficulty discovering novel antibiotics due to data issues	(Ali et al. 2023; Liu et al. 2024)
Model interpretability	Ensuring transparency and understandability of AI models is essential. Some models, being complex, pose challenges in interpreting their decisions	Interpret decisions made by complex AI models	(Ali et al. 2023)
Generalizability	AI models developed in one setting may not be applicable to other contexts, making it challenging to generalize their effectiveness	AI models have limited generalizability to different settings	(Rabaan et al. 2022)
Regulatory and ethical issues	Using AI in drug resistance management raises regulatory and ethical concerns, including data privacy, informed consent, and liability	There is a need to address ethical data privacy and consent issues	(Rabaan et al. 2022)
Cost and infrastructure	The cost of developing and implementing AI models can be high, and not all settings may have the necessary infrastructure to support these models	Developments derive high costs and supportive infrastructure	(Rabaan et al. 2022)

## AI models for antibiotic optimization and recombination

AI models have shown significant promise in optimizing antibiotic combinations and recombination, as well as in the discovery of novel antibiotics. These models leverage ML and deep learning approaches to analyze large datasets and predict the effectiveness of drug combinations, identify new antibiotics, and optimize the production of recombinant proteins (Stokes et al. 2020). In this context, AI can potentially revolutionize the development of antimicrobial drug molecules, offering an intelligent approach to address AMR's challenges. In addition, AI has been employed to search the human genome for microbe-fighting peptides and to predict additional molecules with desired antibiotic properties (Talat and Khan 2023). While these advancements are promising, it is important to note that the success of AI-guided antibiotic discovery relies heavily on the coupling of ML with experimental validation. Therefore, ongoing research and development in this field are essential to fully harness the potential of AI in addressing the global challenge of drug resistance (Liu et al. 2024).

Data collection and pre-processing are the first steps in drug optimization. Whole-genome sequences (WGS) and single-nucleotide polymorphisms (SNPs) with associated phenotypes comprise the bulk of the data (Liu et al. 2020; Ren et al. 2022). In a study (Ren et al. 2022), WGSs were performed with *E. coli* isolates from clinical samples and animals. The data and information were both collected privately and made publicly available online. Several antibiotics were examined, including ciprofloxacin, cefotaxime, ceftazidime, and gentamicin, and the data included susceptible and resistant isolates. Due to the challenge of using complete genome strains, dividing sequences into lengths of k, known as k-mers, are generated as features (Liu et al. 2020), and by identifying interesting insights into small sequences responsible for resistance (Ali et al. 2023), small-length k-mers can be used to identify notable insights into resistance mechanisms.

The next step is the data pre-processing and extracting the features. The final SNP matrix can be built after obtaining reference alleles, variant alleles, and their positions. SNPs can be encoded as chaos games (CGRs) (A, G, C, T, and N), label encodings, or one-shot encodings to train the ML models. For instance, a, G, C, T, and N may be encoded as 1, 2, 3, 4, or 0 in the SNP matrix (Liu et al. 2020). Labels with phenotypes are also assigned to k-mers for encoding (Rabaan et al. 2022). Multiple Python packages can be used to perform data pre-processing, encoding, and feature extraction (Aslam et al. 2021b). Essential features can also be generated with the help of ML and statistical tools. For instance, a CNN (convolutional neural network) with ML models has developed interesting features to predict AMRs (Kuang et al. 2022).

Regarding the management, classification, and prediction of AMRs, different ML models have been utilized (Liu et al. 2020), such as SVM, RF, CNN, and logistic regression. Similarly, the researchers used a deep learning model comprising artificial neurons layers mimicking the human brain (Arango-Argoty et al. 2018; Li et al. 2021). The scikit-learn Python library implements LR, RF, and SVMs, whereas CNN and other deep learning architectures can be applied with Python and TensorFlow (Aslam et al. 2021b). These models are based on available data to generate a mathematical relationship between target labels and input features. As a result, selecting relevant data is crucial. The models can use the training data repeatedly to map and learn the underlying relationships (Aslam et al. 2023).

Before models can be applied practically, they must be tested against test data (unseen data), for performance validation. Various evaluation metrics can be used to evaluate models, including accuracy, confusion matrix, mean absolute error (MSE), precision, recall, root mean square error (RMS), among others (Hicks et al. 2022; Aslam et al. 2023). Once accuracies are satisfied, it can be applied for practical purposes.

### AI algorithms for rapid antimicrobial susceptibility testing

AI algorithms are being developed for rapid antimicrobial susceptibility testing, offering a potential solution to the slow and labor-intensive traditional methods. For instance, researchers at the University of Oxford have used a combination of fluorescence microscopy and AI to detect antibiotic resistance, achieving at least 80% accuracy on a per-cell basis. Similarly, other studies have highlighted the potential of AI in rapidly testing antimicrobial susceptibility and identifying resistant strains within 30 min (Tran et al. 2022). These AI-based approaches have the potential to significantly reduce the time required for antimicrobial susceptibility testing, offering a promising solution to the challenges posed by traditional diagnostic methods (Anahtar et al. 2021).

Antimicrobial susceptibility testing (AST) is crucial in AI-driven healthcare. Day Zero Diagnostics Inc. (DZD), a company specializing in infectious disease diagnostics, employs whole-genome sequencing and AI to combat the escalating problem of antibiotic-resistant infections. They have devised an AI-powered algorithm known as Keynome® g AST, which utilizes whole-genome sequences to forecast the antimicrobial susceptibility of infections. DZD’s groundbreaking work has earned them two presentations at the 2023 ID Week conference, where they will unveil their AI-driven models for predicting antimicrobial susceptibility. Their mission is to revolutionize the diagnosis and treatment of infectious diseases by swiftly determining the infecting species and the infection’s susceptibility to antimicrobial agents directly from clinical samples, obviating the need for traditional cultures. AI algorithms excel at processing vast datasets and revealing intricate patterns that may elude human researchers. This capability leads to more accurate and efficient predictions, particularly regarding drug resistance. Implementing AI to anticipate drug resistance patterns can transform disease management, enabling personalized treatment strategies tailored to individual genetic and disease attributes.

### The Naïve Bayes (NB) algorithm

The Naïve Bayes (NB) algorithm is used in the context of drug resistance to predict effective drug combinations. The NB algorithm works on the principle of conditional probability, assuming that a particular feature in a class is independent of the presence of any other feature. This assumption makes the algorithm simple, fast, and accurate, suitable for large continuous and discrete datasets. The algorithm calculates the probability of a specific event based on prior knowledge of conditions, and it is widely applied in various fields, including text classification, face recognition, and medical diagnosis. NB classifiers are part of a family of generative learning algorithms known for their simplicity, ease of implementation, and ability to handle continuous and discrete data (Bowers et al. 2006).

A study described an improved naïve Bayesian algorithm to predict effective drug combinations, which can be more productive and less vulnerable to adaptive drug resistance. The algorithm considers drug targets, pathways, side effects, metabolic enzymes, and drug transporters to construct classification models for predicting effective drug combinations (Bai et al. 2018). Another study mentioned the NB model is widely used for classification and is particularly suitable when the input dimensionality is high (Abd El-Hafeez et al. 2024). The improved NB algorithm outperformed the traditional algorithm, which has been used to detect dechallenge, a response observed for the reduction or disappearance of adverse drug reactions (ADR) on withdrawal of a drug (Banu et al. 2014). Researchers used NB and Apriori algorithms to determine the main resistance factor and extract resistant patterns (Rezaei-hachesu et al. 2018). In parallel, another researchers group utilized NB to estimate the probabilities of ineffective treatment due to AMR (Choisy et al. 2019). In this respect, a review of AI applications for AMR mentions using NB and Apriori algorithms to determine the key factors of resistance and extract resistant patterns (Lv et al. 2021). Therefore, the NB algorithm is utilized in drug resistance research to predict effective drug combinations and determine key resistance factors.

### Decision tree

The Decision Tree (DT) algorithm is usually used to predict drug resistance and its classification and improve mutation prediction (Yurtseven et al. 2023). A simple decision tree can define three classes. Decision trees learn in three steps: feature selection, tree generation, and tree pruning (Saraswat 2022). ID3, C4.5, and CART provide the primary foundations for these steps (Quinlan 1979; Aaai/Iaai 1996; Breiman et al. 2017); as a result of using the DT model to estimate the burden of AMR, appropriate medical resources can be allocated (Naylor et al. 2017, 2018). According to Reynolds et al., reducing AMR or improving antibiotic selection can save healthcare utilization and costs (Reynolds et al. 2014). In another study, DT models based on procalcitonin (PCT) were used to guide antibiotic use, which led to shorter treatment durations and smaller total antibiotic doses (Schuetz et al. 2012; Voermans et al. 2019). Researchers developed a DT approach called Treesist-TB for predicting treatment resistance to TB drugs based on drug targets, pathways, side effects, and metabolic enzymes (Deelder et al. 2022). Another study used a DT approach to predict the AMR of pathogens causing urinary tract infections. The study used patient descriptors such as prior antibiotic exposures, prior microbiology antibiotic susceptibility data, basic laboratory values, and comorbidities to predict antibiotic resistance in UTI infections (Tandan et al. 2019). The DT algorithm has also been used in predicting AMR in general, with one study reporting that ML methods using genomic and transcriptomic features can offer extraordinary AMR assignment proficiencies (Yasir et al. 2022).

### Random forest

RF is a group of algorithms that employs multiple Decision Trees to predict drug resistance and antibiotic combinations and improve accuracy, reliability, and mutation prediction (Liaw and Wiener 2002). Rather than averaging the predictions of all trees (i.e., forest), it uses two key concepts. There are two ways to sample data from the training set: randomly and repeatedly. In addition, a random subset of features may reduce the performance of a single tree in an RF. RFs converge to lower generalization errors with increasing trees (Breiman 2001). Based on chemogenomics data and orthology, Chandrasekaran et al. devised an RF model for predicting antibiotic combination therapy effectiveness (Chandrasekaran et al. 2016), which has a simple and regular structure, low computational complexity (n2/2), and high generalization capability (AUC for synergy = 0.79). However, due to inadequate chemogenomics data (Nichols et al. 2011), Mason et al. improved the chemogenomics data using the molecular fingerprint. Mason et al. employed the molecular fingerprint as a feature to enhance the models’ predictive power (Mason et al. 2017). Another study used the RF algorithm to predict multidrug-resistant TB in Pakistan. The study found that RF outperformed all other algorithms and recommended using 20 of the considered total number of features for disease prediction (Ali et al. 2021). Furthermore, genome sequence-based RF models were developed to predict antimicrobial minimum inhibitory concentrations (MIC) of nontyphoidal Salmonella in Taiwan in 2023. Study findings revealed that the proposed RF algorithm is a non-linear learning method that uses 10-mer-based ensemble rules for prediction (Wang et al. 2023).

### Support vector machine

Support Vector Machine (SVM) is a binary classification model for prediction and classification that identifies a partitioning hyperplane in the sample space to separate samples into different classes (Awad and Khanna 2015).

SVM models have often been used to predict AMR phenotypes, e.g., ‘resistant’ or ‘susceptible’ (Her and Wu 2018; Liu et al. 2020). For instance, Her et al. used SVM models to predict whether *E. coli* is resistant to antibiotics. Based on the AUC, this model could make average predictions up to 0.95 accurate (Her and Wu 2018). A study conducted by Liu et al. examined the resistance to five drugs (ampicillin, enrofloxacin, sulfisoxazole, tetracycline, and trimethoprim) using SVM models (Liu et al. 2020), which were found to be 90% or greater accurate. Therefore, SVM models are potential tools for AMR surveillance and clinical diagnosis. Different studies developed a method called SVMMDR to predict the association between miRNA and drug resistance and used SVM to predict treatment failure and drug resistance in TB patients. SVM has shown promising performance in predicting drug resistance and treatment outcomes, making it a valuable tool in drug resistance management (Kanesamoorthy and Dissanayake 2021; Duan et al. 2022).

### Artificial neural network

Artificial Neural Networks (ANNs) are used in drug resistance management for various applications, including drug repurposing testing and AMR prediction. ANNs, a subset of ML, are inspired by the human brain and can recognize patterns and learn from examples, making them valuable for analyzing complex datasets and making predictions. In drug resistance, ANNs have been used for drug repurposing testing by screening known substances for antimicrobial effects and studying interactions between substances and bacterial structures. In addition, ANNs have been applied to predict antibiotic resistance, offering a valuable tool for combating high AMR rates. The use of ANNs in drug resistance management demonstrates the potential of AI in addressing the challenges of AMR and accelerating the development of new methods to combat drug resistance (Song 2021; Jukič and Bren 2022; Rabaan et al. 2022; Popa et al. 2022; Deelder et al. 2022). Through a deep learning approach, Stokes et al. found new antibiotics without making assumptions. These molecules have inhibited various pathogens in mouse experiments. As a next step, we will use deep learning to design new antibiotics and optimize existing molecules. This phase could represent a paradigm shift in antibiotic discovery in the future (Stokes et al. 2020). Table 3 comprises different ML algorithms in drug resistance.

**Table 3:** Comparison of machine learning algorithms in drug resistance

Algorithm	Description	Applications	Examples/results	Source
Naïve Bayes (NB)	Predicts drug resistance patterns using conditional probability and assuming feature independence. NB is known for simplicity and speed	Drug resistance prediction, effective drug combination, dechallenge	Improved NB algorithm predicts effective drug combinations with enhanced efficacy The NB model excels in high input dimensionality classification NB estimates probabilities of ineffective treatment due to AMR	(Bowers et al. 2006; Banu et al. 2014; Bai et al. 2018; Rezaei-hachesu et al. 2018; Choisy et al. 2019; Lv et al. 2021; Abd El-Hafeez et al. 2024)
Decision Tree	Predicts drug resistance and guides antibiotic use. Decision Tree utilizes feature selection, tree generation, and pruning. Its customized approaches enhance accuracy	Drug resistance prediction, antibiotic selection, mutation prediction	Decision Trees allocate medical resources by estimating AMR burden Procalcitonin-based Decision Trees shorten treatment duration Treesist-TB predicts TB drug resistance	(Quinlan 1979; Aaai/Iaai 1996; Schuetz et al. 2012; Reynolds et al. 2014; Naylor et al. 2017, 2018; Breiman et al. 2017; Tandan et al. 2019; Voermans et al. 2019; Saraswat 2022; Yasir et al. 2022; Deelder et al. 2022; Yurtseven et al. 2023)
Random Forest	RF is an ensemble algorithm using multiple decision trees to predict drug resistance. It employs random sampling and feature subset selection. RF demonstrates high accuracy	Drug resistance prediction, antibiotic combination, AMR burden	RF predicts effective antibiotic combinations with high accuracy RF outperforms other algorithms in predicting multidrug-resistant TB	(Breiman 2001; Liaw and Wiener 2002; Nichols et al. 2011; Chandrasekaran et al. 2016; Mason et al. 2017; Ali et al. 2021; Wang et al. 2023)
Support Vector Machine	SVM offers a binary classification model for predicting AMR phenotypes. It provides high accuracy in *E. coli* antibiotic resistance prediction. SVM is valuable in drug resistance management	AMR phenotype prediction, antibiotic resistance prediction	SVM accurately predicts antibiotic resistance in *E. coli* SVM predicts miRNA–drug resistance associations and TB treatment outcomes effectively	(Awad and Khanna 2015; Her and Wu 2018; Liu et al. 2020; Kanesamoorthy and Dissanayake 2021; Duan et al. 2022)
Artificial Neural Network	ANN utilizes Artificial Neural Networks for drug repurposing testing and antibiotic resistance prediction. It recognizes patterns and learns from examples. It is applied to discover new antibiotics	Drug repurposing testing, antibiotic resistance prediction	ANNs identify new antibiotics and predict antibiotic resistance ANNs offer potential in combating AMR	(Stokes et al. 2020; Song 2021; Jukič and Bren 2022; Rabaan et al. 2022; Popa et al. 2022; Deelder et al. 2022)

## Real-time AI applications in drug resistance management

Numerous software tools are accessible for managing drug resistance, each offering unique capabilities.

*DrugRepo* is a computational pipeline for repurposing drugs for new indications, including various cancers, kidney diseases, and cardiovascular. DrugRepo uses a multi-step process, such as compound–target data analysis, structural analysis, gene–disease relationships, and pathway analysis. The pipeline can repurpose approximately 0.8 million compounds across 606 diseases (Wang et al. 2022). DrugRepo is not designed explicitly for drug-resistance management, but it can be used to repurpose drugs for new indications, which may include drug resistance-related applications. For example, DrugRepo could identify existing drugs that can be repurposed to treat drug-resistant infections or improve the efficacy of existing treatments. However, additional ML algorithms and techniques would be required to address drug resistance specifically and analyze and predict drug resistance patterns (Liu et al. 2021; Deelder et al. 2022; Dróżdż et al. 2023).

**ENDS:** The Epistemic Nonparametric Drug-response Scoring (ENDS) is an online tool that presents non-parametric models for the curve fitting and scoring drug dose–responses. A linear fit of the drug sensitivity is not subjected to any parametric assumptions. As a result, parallel indexing can be performed, such as half-maximal inhibitory concentrations (IC50) and area under curve (AUC). A non-parametric model such as a spline (npS), a monotonic model, and a Bayesian model is provided in the tool, along with other indices such as a maximum effective dose and a gradient of drug–response span. With the ENDS tool, drug sensitivity can be more accurately and less biasedly assessed, allowing for a more accurate analysis of drug response (Amiryousefi et al. 2022).

While the ENDS tool is not designed explicitly for drug resistance management, it can be used to assess drug responses, which is relevant to understanding and managing drug resistance. By providing a more objective and less biased assessment of drug responses, the ENDS tool can contribute to the broader efforts in drug resistance management by enabling a more accurate understanding of how drugs interact with pathogens and how they may lead to or mitigate drug resistance (Deelder et al. 2022).

## Conclusion and future research

The study highlights remarkable progress in AI applications, from creating new antibiotics to foreseeing AMR patterns. AI enables crafting personalized treatment plans tailored to each person’s unique genetic makeup and illness traits. Furthermore, AI algorithms have played a crucial role in fine-tuning antibiotic combinations, predicting resistance, and speeding up antimicrobial testing. Yet, despite these advancements, hurdles remain. Challenges like ensuring data quality, making models more straightforward to understand, and addressing ethical concerns need our attention to fully leverage AI’s potential in fighting drug resistance. This review emphasizes how AI transforms our global approach to combating drug resistance. This paper is a valuable guide for researchers, healthcare providers, policymakers, and educators in this battle by sharing success stories, discussing ethical considerations, and charting future paths. A multi-sectoral approach involving infection control, antibiotic accessibility, and robust surveillance is crucial to address AMR.

Adopting a multi-sectoral approach, as recommended by the CDC and WHO, to combat AMR is crucial. The CDC stresses the importance of setting goals across various sectors, including healthcare, food, communities, and the environment, and implementing a comprehensive plan using the One Health approach. This plan involves infection prevention and control, improving antibiotic accessibility, establishing robust data tracking systems, and enhancing laboratory capacity for identifying resistant bacteria. Similarly, the WHO emphasizes the need for solid national action plans, improved surveillance of antibiotic-resistant infections, and the implementation of infection prevention and control measures. They also offer educational modules for healthcare workers. Furthermore, research suggests that providing healthcare professionals with surveillance data can drive behavioral changes at individual and organizational levels, as seen in initiatives like the UK Five Year AMR Strategy. These collective recommendations underscore the importance of a global effort that spans healthcare, surveillance, education, and behavior change to address AMR effectively.

Predicting drug resistance patterns encounters various obstacles, mainly due to the scarcity of reliable data. AI algorithms rely on extensive datasets to make accurate predictions, but such data is often lacking. Moreover, ethical concerns surrounding AI in healthcare, such as worries about data privacy and security and the potential for AI to worsen existing healthcare disparities, add another layer of complexity. To address these challenges and improve the accuracy of drug resistance predictions, researchers are pioneering innovative techniques that offer both precision and transparency. For example, a recent study introduced a novel method inspired by group testing and Boolean compressed sensing, which yields highly accurate and interpretable results. This approach is flexible, allowing for optimization across various evaluation metrics simultaneously.

In addition, researchers are exploring new data collection and analysis methods using AI for decision-making processes. AI algorithms excel at processing large datasets and uncovering hidden patterns that may elude human perception, thus enhancing the accuracy and efficiency of drug resistance prediction. Integrating AI into drug resistance forecasting holds immense promise for transforming disease management, as it enables the development of personalized treatment plans tailored to each patient’s unique genetic profile and disease characteristics.

However, predicting drug resistance patterns faces challenges due to data scarcity and ethical concerns. Integrating AI offers promising solutions, enhancing accuracy and enabling personalized treatment plans. With ongoing research and collaboration, AI promises to revolutionize disease management and usher in more effective strategies against drug resistance.

## Data Availability

Data sharing is not applicable to this article as no datasets were generated or analyzed during the study.
